# Plasma volume expansion reveals hidden metabolic acidosis in patients with diabetic ketoacidosis

**DOI:** 10.1186/s40635-022-00464-5

**Published:** 2022-08-29

**Authors:** Robert Svensson, Robert G. Hahn, Joachim H. Zdolsek, Hans Bahlmann

**Affiliations:** 1 Department of Anesthesiology and Intensive Care, Linköping University, Vrinnevi Hospital, Norrköping, Sweden; 2 Department of Biomedical and Clinical Sciences, Linköping University, Linköping, Sweden; 3 Research Unit, Södertälje Hospital, 152 86 Södertälje, Sweden; 4 Karolinska Institutet at Danderyds Hospital (KIDS), Stockholm, Sweden; 5 Present Address: Department of Anesthesiology and Intensive Care, Linköping University, University Hospital, Linköping, Sweden

**Keywords:** Diabetic ketoacidosis, Acid–base balance, Water–electrolyte balance, Dehydration, Fluid therapy, Blood volume

## Abstract

**Background:**

Hyperchloremic metabolic acidosis that develops during the treatment of diabetic ketoacidosis is usually attributed to the chloride content of resuscitation fluids. We explored an alternative explanation, namely that fluid-induced plasma volume expansion alters the absolute differences in the concentrations of sodium and chloride (the Na–Cl gap) enough to affect the acid–base balance. We analyzed data from a prospective single-center cohort study of 14 patients treated for diabetic ketoacidosis. All patients received 1 L of 0.9% saline over 30 min on two consecutive days. Blood gases were sampled before and after the infusions.

**Results:**

The initial plasma volume was estimated to be 25 ± 13% (mean ± SD) below normal on admission to the intensive care unit. At that time, most patients had an increased actual Na–Cl gap, which counteracts acidosis. However, the correction of the plasma volume deficit revealed that these patients would have had a decreased Na–Cl gap upon admission if they had been normovolemic at that time; the estimated “virtual Na–Cl gap” of 29 ± 5 mmol/L was significantly lower than the uncorrected value, which was 39 ± 5 mmol/L (*P* < 0.001). On Day 2, most patients had a decreased actual Na–Cl gap (33 ± 5 mmol/L), approaching the corrected value on Day 1*.*

**Conclusions:**

The hyperchloremic acidosis commonly seen in diabetic ketoacidosis may not be primarily caused by the chloride content of resuscitation fluids but, rather, by the restoration of plasma volume, which reveals the hidden metabolic acidosis caused by a decreased Na–Cl gap.

*Trial registration* Clinical Trials Identifier NCT02172092, registered June 24, 2014, https://www.clinicaltrials.gov/NCT02172092

**Supplementary Information:**

The online version contains supplementary material available at 10.1186/s40635-022-00464-5.

## Background

Patients with diabetic ketoacidosis (DKA) present with metabolic acidosis, which is assumed to be due to the accumulation of ketoacids and, sometimes, lactate. The treatment of DKA includes infusion with isotonic saline. The surplus of chloride ions in this fluid is believed to cause hyperchloremic acidosis, which makes the metabolic acidosis difficult to reverse [1]. Alternatively, the development of hyperchloremic acidosis during the treatment of DKA is explained by renal retention of chloride due to a lack of bicarbonate [2] or a preferential excretion of ketones [3].

According to the Stewart approach to acid–base disorders, chloride (Cl) affects acidity by being the principal negative ion component of the “strong ion difference” (SID). The main positively charged component is sodium (Na). Acidosis develops if the difference between [Na^+^] and [Cl^−^] (Na–Cl gap) is decreased to below normal, which is approximately 38 mmol/L [4]. Such a decrease in SID can result from the infusion of fluid with a small or non-existent Na–Cl gap, such as saline. A similar decrease in SID is achieved by increasing the water content of the plasma because SID is related to *absolute* differences between plasma electrolyte concentrations. For example, if plasma with a sodium concentration of 140 mmol/L and a chloride concentration of 100 mmol/L (Na–Cl gap 40) is diluted by 10%, the new sodium concentration will be 127 mmol/L, and the new chloride concentration will be 91 mmol/L. The resulting Na–Cl gap is 36, which represents a reduction of SID.

Plasma volume is invariably decreased in severe DKA. Mathematically, this will *increase* the Na–Cl gap and counteract acidosis. An alternative but older explanation for this phenomenon of “contraction alkalosis” is that the plasma bicarbonate concentration increases when water is lost from the extracellular fluid volume [5].

The intravenous fluid therapy that is mandatory in DKA increases the water content of the extracellular fluid, which, in itself, decreases the Na–Cl gap. However, the summary effect of fluid therapy is complex because an infusion dilutes the concentrations of weak and strong acids and may also decrease the lactate concentration by improving tissue perfusion. Such events could potentially compensate for the decreasing Na–Cl gap.

The purpose of the present study was to outline the effect of plasma volume expansion on acid–base status during the treatment of DKA in intensive care patients.

## Methods

The present analysis is a prespecified secondary publication of a prospective non-randomized clinical study of fluid therapy in DKA [6]. The specific aim of this study was to outline the effect of plasma volume expansion on acid–base status during the treatment of DKA.

Seventeen adult patients with DKA, clinically defined as ICU-requiring severe hyperglycemia, who were admitted to the intensive care unit (ICU) of Vrinnevi Hospital, Norrköping, Sweden, were enrolled in a study analyzing the distribution of infusion fluids. The study was approved by the Regional Ethics Committee of Linköping (2014/123–31) and registered at clinicaltrials.gov (NCT02172092). Each patient provided oral and written consent for participation. Fourteen patients participated in the experiment on both study days, and only these patients were used in the present analysis.

### Fluid therapy and measurements

After arrival in the ICU, 1 L of 0.9% saline was infused intravenously over 30 min (Day 1), which was repeated on the day after admission (Day 2). Further fluid resuscitation was withheld until 30 min after the first infusion, then using Ringer’s acetate.

Arterial blood was sampled every 10 min from the start of the saline infusion. We report the biochemical data obtained just before the infusion started and at the infusion. Blood pH, P_a_CO_2_, base excess (BE) and the plasma concentrations of bicarbonate (HCO_3_) glucose, sodium (Na), potassium (K), and lactate were analyzed using a Radiometer ABL 800 FLEX blood gas machine (Radiometer Medical, Copenhagen, Denmark). The hematocrit (Hct) and the plasma concentrations of chloride (Cl), magnesium (Mg), calcium (Ca) and albumin were measured on a COBAS 8000 platform (Roche Inc., Mannheim, Germany).

The patients had received a bladder catheter through which the excreted urine was collected, measured, and sampled at 3 h. The urinary concentrations of Na, Cl, K, Mg, and glucose concentrations were measured on the COBAS 8000. The excretion of electrolytes and glucose was taken as the product of the urine volume and the urinary concentration of the solute in question.

### Calculations

We compared plasma variables at four points in time, which were denoted *t*_1_ to *t*_4_. They represented *t*_1_ = before the infusion on Day 1, *t*_2_ = at the end of the infusion on Day 1, *t*_3_ = before the infusion on Day 2, and *t*_4_ = at the end of the infusion on Day 2. At *t*_3_, patients had been fluid resuscitated since the day before, and therefore patients were considered to have normal plasma volume at this time. Hence, the Hb concentration and the Hct measured at *t*_3_ were considered to reflect intravascular normovolemia (“late baseline”). Total erythrocyte volume was assumed to be constant.

The relative change in plasma volume (ΔPV) before the first saline infusion as compared to the late baseline reflected the hypovolemia that was present on arrival at the ICU and was calculated as follows:

$$\Delta {\text{PV}} = 1{-}\left[ {\left( {{\text{Hb}}_{3} /{\text{Hb}}_{1} } \right) \times \left( {1 - {\text{Hct}}_{1} } \right)/\left( {1 - {\text{Hct}}_{3} } \right)} \right]$$

where the terms that include Hct convert hemodilution into plasma dilution. The ΔPV caused by the infusions was calculated in the same way, but then comparing the Hb and Hct measured just before and at the end of the infusion.

To define a normal Na–Cl gap, normal [Na^+^] was defined as the middle of the local laboratory reference interval (137–145 mmol/L), i.e., 141 mmol/L. Likewise, normal [Cl^−^] was defined as the middle of the laboratory reference interval (98–109 mmol/L), i.e., 104 mmol/L. The normal Na–Cl gap was thus calculated as 37 mmol/L.

We calculated the virtual Na–Cl gap at *t*_1_ that would be expected if patients had been normovolemic upon admission to the ICU (at time *t*_1_). This was done by restoring PV to the “late baseline” (time *t*_3_) by infusing a theoretical volume of water. The virtual Na–Cl gap was then obtained as follows:

$$^{\prime\prime}{\text{Virtual}}^{\prime\prime}{\text{ Na - Cl gap}}, \, t_{1} = \left( {P{\text{ - Na}}_{1} {-}P{\text{ - Cl}}_{1} } \right) \times (1{-}\Delta{\text{PV}})$$

“Other ions” (OI) were calculated as described by Story [7], slightly modified for local normal values. Measured base excess is partitioned into changes in sodium and chloride, lactate, albumin and other ions as compared to normal. Hence, OI = BE – ([Na^+^] – [Cl^−^] – 37) – (1.5 – [lactate]) – (0.25 (42 – [albumin])).

### Statistics

Results are expressed as mean ± SD unless otherwise noted. Normality was assessed using the Kolmogorov–Smirnov test. Differences between measurements were evaluated using a Student’s *t-*test or the Wilcoxon signed-rank rank test for related samples, as appropriate. Occasional missing values were imputed by taking the mean of the two most adjacent values. Statistica version 13.5.0.17 (TIBCO Software Inc., Palo Alto, California, USA) was used for statistical analyses.

## Results

The patient data were collected between 2014 and 2019. The mean age of the finally included 14 patients was 51 years (range 18–86), and their body weight averaged 73 kg (range 55–92 kg). Original data are provided in Additional file 1.

A bedside test for ketones in capillary blood resulted in a concentration of 5.2 ± 2.0 mmol/L upon arrival at the emergency department. The first glucose concentration in arterial plasma obtained in the ICU was 35 ± 9 mmol/L.

**Fig. 1:**
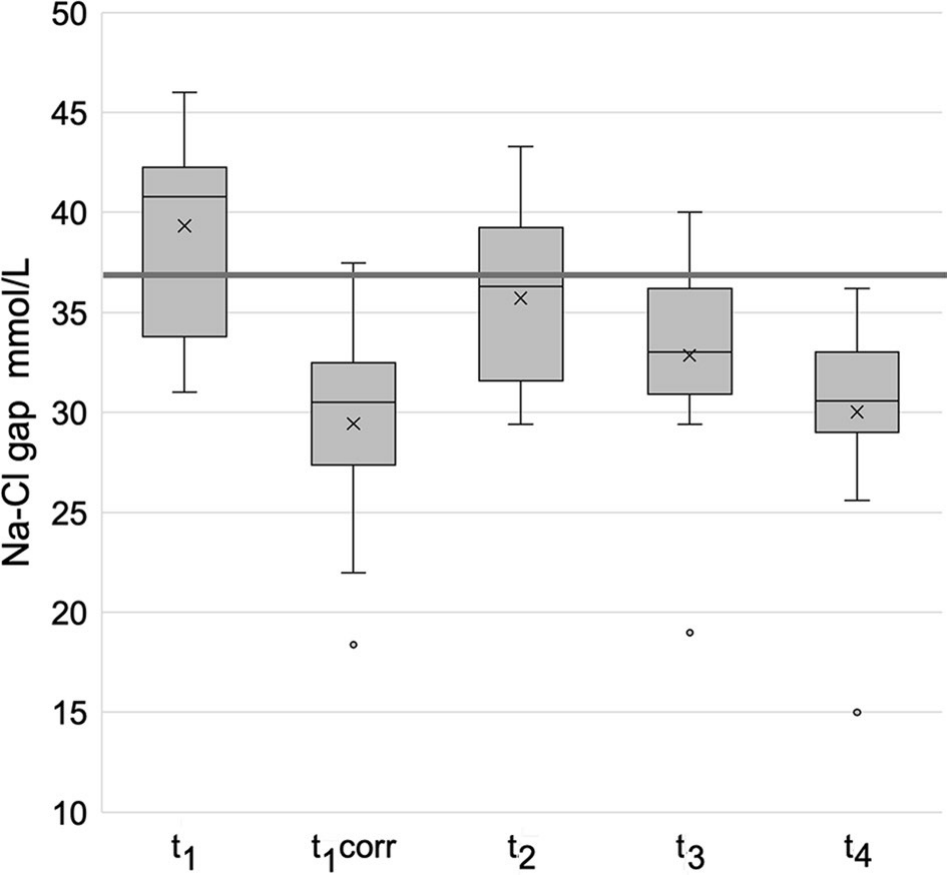
Distribution of Na–Cl gap values. Boxplot showing values for the Na–Cl gap during the experiment. *t*_1_ at admission to intensive care, *t*_1corr_ the same values after correction for hypovolemia, *t*_2_ values at the end of a 1 L infusion of 0.9% saline over 30 min on Day 1, *t*_3_ and *t*_4_ values before and just after infusion of another liter of saline on Day 2. At *t*_3_, patients were considered to be normovolemic. Horizontal bar represents the Na–Cl gap defined as normal, i.e., 37 mmol/L

Upon inclusion, BE was − 16 ± 7 mmol/L, and the actual Na–Cl gap was 39 ± 5 mmol/L. Group data for the Na–Cl gap before and after fluid infusions are shown in Fig. 1, and individual values are shown in Additional file 2.

### Correcting the Na–Cl gap for hypovolemia

Before the first infusion, ten patients had Na–Cl gaps > 37 (increased), and four patients had Na–Cl gaps < 37 (decreased). Thus, most patients presented with an increased Na–Cl gap, causing alkalosis, which partly countered the effect of the metabolic acidosis caused by DKA.

The calculated deficit in plasma volume upon inclusion was estimated to be 25 ± 13%. After correcting for this hypovolemia, as many as 13 out of the 14 patients would have a “virtual” Na–Cl gap below 37 mmol/L; the group value would be 29 ± 5 mmol/L (*P* < 0.001, as compared to the uncorrected value, which was 39 ± 5 mmol/L).

### Na–Cl gap responses to 0.9% saline

On Day 1, the plasma volume increased by 16 ± 7%, while the Na–Cl gap decreased by 4 ± 3 mmol/L in response to the infusion of 1 L of saline. However, BE did not change, due to concomitant dilution of albumin, lactate, and OI.

The total amount of administered fluid between the beginning of the study on Day 1 and up to the second infusion (on Day 2) amounted to 6.9 ± 1.9 L. The infused fluid consisted of Ringer´s acetate solution except for the experimental infusion on Day 1.

On Day 2, before the saline infusion, the actual Na–Cl gap was below the normal value (37 mmol/L) in 11 of the 14 patients. During the infusion, plasma volume increased by 12 ± 7%, which was no different from the increase on Day 1 (*P* = 0.09). The Na–Cl gap then decreased by a further 3 ± 2 mmol/L. As shown in Table 1, BE decreased significantly during the second infusion because the concomitant fluctuations in the concentrations of albumin, lactate, and OI were small as compared to those on Day 1.

**Table 1:** Biochemical parameters and derived parameters just before and after the saline infusions on Days 1 and 2 (blood) and from just before to 2.5 h after the saline infusions (urine)

	Day 1			Day 2		
	Before	After	*P*	Before	After	*P*
Glucose (mmol/L)	34.8 ± 9.3	31.2 ± 8.2	< 0.001	14.5 ± 2.9	13.9 ± 3.1	0.042
Creatinine (µmol/L)	117 ± 51	114 + 53	0.008	78 + 33	67 ± 29	0.02
pH (pH units)	7.24 ± 0.09	7.23 ± 0.08	0.06	7.40 ± 0.07	7.39 ± 0.08	0.002
PaCO_2_ (torr)	23 ± 9	25 ± 10	0.16	32 ± 6	32 ± 7	0.34
PaCO_2_ (kPa)	3.1 ± 1.2	3.3 ± 1.3	0.16	4.2 ± 0.8	4.3 ± 0.9	0.34
aHCO_3_ (mmol/L)	11 ± 6	11 ± 6	0.87	20 ± 5	19 ± 5	0.019
TCO_2_ (mmol/L) ^1^	11 ± 6	11 ± 6	0.7	21 ± 5	20 ± 5	0.027
BE (mmol/L)	− 16 ± 7	− 16 ± 7	0.83	−4 ± 6	−5 ± 5	0.004
P-K (mmol/L)	5.0 ± 0.6	4.7 ± 0.5	0.002	3.9 ± 0.5	3.7 ± 0.5	0.034
P-Na (mmol/L)	132 ± 6	134 ± 6	0.013	138 ± 6	137 ± 6	0.07
P-Cl (mmol/L)	93 ± 6	98 ± 6	< 0.001	105 ± 7	107 ± 7	< 0.001
∆ Na–Cl (mmol/L)	39 ± 5	36 ± 4	< 0.001	33 ± 5	30 ± 5	< 0.001
Lactate (mmol/L)	1.7 ± 0.6	1.3 ± 0.4	< 0.001	0.9 ± 0.5	0.8 ± 0.4	0.031
Anion Gap (mEq/L)^2^	37 ± 9	33 ± 9	0.001	20 ± 5	18 ± 6	0.006
Albumin (g/L)	40 ± 9	36 ± 8	< 0.001	31 ± 5	27 ± 5	< 0.001
Other ions (mEq/L)	− 18 ± 9	−16 ± 9	0.016	−3 ± 5	−3 ± 5	0.26
Hb (g/L	148 ± 27	136 ± 24	< 0.001	124 ± 21	115 ± 17	< 0.001
Hct (%)	45 ± 8	42 ± 7	< 0.001	38 ± 6	36 ± 5	< 0.001
**Urinary excretion 0–3 h**						
Volume (mL)		893 (650–1180)			360 (250–560)	0.004
Na (mmol)		57 (33–77)			41 (15–52)	0.36
Cl (mmol)		25 (15–51)			50 (20–90)	0.18
K (mmol)		17 (11–23)			11 (7–16)	0.33
Mg (mmol)		1.4 (0.9–1.7)			1.1 (0.7–1.5)	0.68
Glucose (mmol)		148 (100–245)			51 (4–73)	< 0.002

Blood data are reported as mean ± SD and the urine data as the median (25th–75th percentiles)

^1^ TCO_2_ was calculated as [aHCO_3_^-^] (mmol/L) + 0.03 * PaCO_2_ (torr)

^2^ The Anion Gap was calculated as ([Na]+[K]+2[Ca]+2[Mg])-([Cl]+[HCO_3_]+[Lactate])

### Kidney function

Plasma creatinine before the DKA episode was available in 12 patients and was within the normal range in all cases (< 115 µmol/L), but on the high side in three of them (100, 100, and 111 µmol/L). Plasma creatinine was transiently elevated during the hospital episode with a maximum of 117 ± 51 µmol/L on admission to the ICU (Table 1). By contrast, all values were normal during the post-ICU follow-up (66 ± 19 µmol/L).

The urinary excretion of sodium exceeded the chloride excretion during the first infusion experiment (*P* = 0.002), which would decrease the Na–Cl gap, while these electrolytes were excreted in more similar amounts during and after the second infusion (Table 1).

## Discussion

### Key findings

The experimental setup of the study, with controlled fluid infusions and the repeated sampling of acid–base parameters, hemoglobin, and electrolytes, made it possible to reveal the relationship between the acid–base status and relative changes in PV in patients with DKA. The contribution of changes in the Na–Cl gap to metabolic acidosis upon admission appeared to be limited at first sight. Most patients even had an increased Na–Cl gap, which partly countered the acidosis caused by ketoacids. However, after correcting for hypovolemia, almost all patients had a decreased Na–Cl gap. Thus, hypovolemia masks significant deviations in the Na–Cl gap in DKA.

### Clinical implications

Our findings suggest that the commonly described phenomenon of progressive hyperchloremic acidosis during the treatment of DKA may not be primarily caused by chloride-rich resuscitation fluids or by renal retention of chloride. Instead, a masked, low Na–Cl gap is already present initially and is then revealed by the restoration of the intravascular volume.

This phenomenon can be expected to be pronounced when plasma volume is expanded by infusion fluids with a Na–Cl gap of zero, irrespective of the absolute amount of chloride in the solution. According to the Stewart paradigm, an infusion of saline has the same effect on SID, BE, and pH as an infusion of only water because they have the same effect on the Na–Cl gap. In our study, an infusion of saline significantly decreased the Na–Cl gap, but its effect on BE was offset by the dilution of albumin, lactate, and OI.

A simple calculation supports our view that the surplus of chloride in 0.9% saline cannot cause acidosis without a concomitant change in extracellular fluid volume. In the present study, the initial Na and Cl concentrations were 132 and 93 mmol/L, respectively. A volume deficit of 25% indicates that the extracellular fluid volume would be approximately 11 L, and its content in terms of Na and Cl ions would then be 1452 and 1023 mmol, respectively. Using mass balance, the Na–Cl gap will remain at 39 mmol/L, regardless of how many equal amounts of Na and Cl are added, provided that the increased amounts are dispersed in 11 L of fluid.

The use of infusion fluids with a normal or even supraphysiological Na–Cl gap can be expected to mitigate or even compensate for the decrease in the Na–Cl gap caused by plasma volume expansion. This would, at least in theory, support the use of solutions such as Plasma-Lyte 148 (Baxter Inc., Springfield, Illinois, USA) for resuscitation in DKA, even in patients presenting with normal or even increased Na–Cl gaps. We used 1 L of 0.9% saline during the experiments, while Ringer’s acetate was used for resuscitation between the experimental infusions. This initial saline infusion was intended to counteract the high incidence of hyponatremia on admission. However, Ringer’s acetate has a SID of 30 which is still lower than plasma and this infusion fluid is, therefore, not able to correct a low Na–Cl gap.

This also explains why the Na–Cl gap at *t*_3_ (assuming normovolemia) was larger than the “virtual Na–Cl gap” at t_1_. The latter was calculated by hypothetically expanding the PV at *t*_1_ with water until it was equal to the PV at t_3_. This “virtual Na–Cl gap” then quantifies the impact of loss of plasma water per se on the Na–Cl gap or, in other words, the decrease of the Na–Cl gap that can be expected solely by normalizing plasma volume. However, the actual Na–Cl gap between Day 1 and Day 2 changed not only because of PV expansion, which decreased the Na–Cl gap, but also because a resuscitation fluid with a SID higher than 0 (Ringer’s acetate) mitigated the decrease of the gap. Surprisingly, the kidneys initially decreased the gap on Day 1 by excreting relatively more Na than Cl. The summary effect of these factors yielded the actual Na–Cl gap at *t*_3_ being larger than the “virtual Na–Cl gap” at t_1_.

The Stewart approach holds that pH is determined by SID and the plasma concentrations of weak acids and P_a_CO_2_. It provides a clear picture of the intimate relation between changes in serum electrolyte concentrations and changes in pH. This information is not provided by the traditionally used Henderson–Hasselbalch equation which, in our material, would show the reduced metabolic acidosis between Day 1 and 2 but not indicating that the acidosis would be worsened by a decreased Na–Cl gap. The Henderson–Hasselbalch equation is still valid but is, according to the Stewart approach, of minor importance since it only describes how pH and bicarbonate as dependent entities relate to changes in the independent entity P_a_CO_2_.

### Literature

Most studies comparing the rate of correction of the acid–base balance in DKA have found small differences between the use of 0.9% saline and balanced crystalloid fluids for resuscitation, and many of these studies have been observational.

Van Zyl et al*.* reported faster normalization of pH with Ringer’s lactate as compared to saline in a randomized trial with 54 patients; however, the difference did not reach significance [8]. The time required for DKA to resolve was shorter with Plasma-Lyte 148 as compared to saline in a study with 66 pediatric patients, but here as well, the difference did not reach statistical significance [9]. Self et al. reported a speedier resolution of DKA with Ringer’s lactate or Plasma-Lyte 148 in a sub-study of a randomized trial involving 172 adult patients [10]. In a retrospective analysis, Oliver et al. found a faster increase in pH in 84 DKA patients who received fluid resuscitation with Plasma-Lyte instead of saline, but the time to resolution did not differ [11].

Two randomized trials comparing saline with Plasma-Lyte 148 in 45 and 90 patients showed faster normalization of acidosis [1, 12]. These findings, as well as a reduced length of stay in patients treated with Plasma-Lyte 148, were recently confirmed in a meta-analysis [13]. Unfortunately, specific data on the course of the Na–Cl gap are lacking in these reports, and a larger trial is needed to establish whether Plasma-Lyte 148 is beneficial in DKA.

### Other contributors to the acid–base balance

The changes in acid–base balance between Day 1 and 2 are not only related to the composition of the resuscitation fluids used, but also internal factors, such as renal homeostasis. Moreover, metabolic changes affecting SID during treatment for DKA are not limited to changes in the Na–Cl gap. The concentrations of other strong ions, such as potassium and lactate, will also change, though to a lesser degree. Blood ketones (which are negative anions) were only measured on admission to the ICU but can be assumed to have decreased during treatment. This is reflected in the substantial decrease in other ions in the cohort, as well as in decreases in the strong ion gap (SIG), as reported by others [14, 15].

Most patients presented with elevated plasma albumin, although a few showed low values, which resulted in a low-normal average for the entire cohort. Hypoalbuminemia was common after the plasma volume had been restored, which at least temporarily contributes to the decrease in metabolic acidosis because albumin is a weak acid [16].

### Limitations

The number of included patients was small, and some patients could have received small amounts of fluids before admission to the ICU. Sodium was analyzed using a direct ion-selective electrode (ISE) method. However, this method was not available for chloride when the present study was performed, which forced us to rely on the indirect ISE method. This method involves dilution, whereby measurement errors can occur due to abnormal plasma concentrations of proteins and lipids [17]. This could also explain why our “normal” Na–Cl gap was slightly higher than those usually described. Blood gas analyzers generally report slightly higher chloride values as compared to standard electrolyte analyzers, resulting in smaller normal values for the Na–Cl gap [18].

The use of absolute numbers instead of ranges for normal values for electrolytes and albumin can be questioned. Electrolyte concentrations can fluctuate slightly around the normal value without being considered pathological. However, the actual normal range for SID (and, thus, its principal component, the Na–Cl gap) is much smaller than theoretically possible based on the normal ranges of sodium and chloride [19]. Further, according to the Stewart paradigm, every mmol/L increase or decrease in the Na–Cl gap will cause a concomitant change in BE of 1 mmol/L.

We are aware that the equation used for the calculation of OI that is described by Story [7] is an approximation. For example, this equation does not account for the fact that the albumin charge slightly changes with pH [20]. However, we have no reason to suspect that these limitations significantly affect our findings.

Our calculations assume that erythrocyte volume is constant throughout the experiment and that the plasma volume at t_3_ reflects intravascular normovolemia. We did not measure total urinary excretion of electrolytes such as sodium and chloride, which could have provided more information on the renal contribution of the acid–base status.

## Conclusions

A low initial Na–Cl gap that causes acidosis in patients with DKA is masked by a low plasma volume. Volume expansion unmasks this electrolyte disorder, which makes the reversal of metabolic acidosis more difficult. Our findings present a novel explanation for the commonly observed development of hyperchloremic acidosis during the treatment of DKA.

## Supplementary Information

**Additional file 1.** Individual demographic and biochemical data for the 14 studied patients with diabteic ketoacidosis.

**Additional file 2.** Individual data for the Na-Cl gap during the study. t_1_ at admission to intensive care, t_1corr_ the same values after correction for hypovolemia, t_2_ values at the end of a 1 liter infusion of 0.9% saline over 30 min on Day 1, t_3_ and t_4_ values before and after infusion of another liter of saline on Day 2. At_3_t, patients were considered to be normovolemic. Horizontal bar represents the Na-Cl gap defined as normal, i.e., 37 mmol/L.

## Data Availability

All data generated or analyzed during this study are included in this published article and its supplementary information files.

## Abbreviations

BE: Base excess
DKA: Diabetic ketoacidosis
Hb: Hemoglobin
Hct: Hematocrit
ICU: Intensive care unit
ISE: Ion-selective electrode
OI: Other ions
PV: Plasma volume
SID: Strong ion difference
