# Supplementary material for: Plasma volume expansion reveals hidden metabolic acidosis in patients with diabetic ketoacidosis
Source: Intensive Care Med Exp. 2022 Aug 29;10:36. doi: 10.1186/s40635-022-00464-5 (PMC9424448; doi:10.1186/s40635-022-00464-5)
Supplement: Supplementary file 2 — Additional file 2. Individual data for the Na-Cl gap during the study. t1 at admission to intensive care, t1corr the same values after correction for hypovolemia, t2 values at the end of a 1 liter infusion of 0.9% saline over 30 min on Day 1, t3 and t4 values before and after infusion of another liter of saline on Day 2. At3t, patients were considered to be normovolemic. Horizontal bar represents the Na-Cl gap defined as normal, i.e., 37 mmol/L. [file 40635_2022_464_MOESM2_ESM.pdf]

Supplemental Data File 3. individual Na-Cl values.

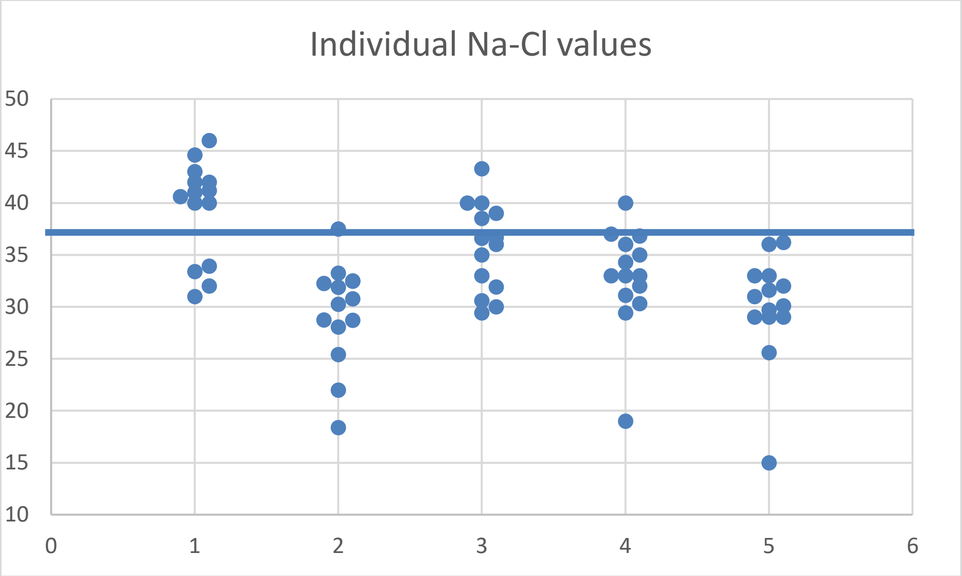

Individual values for the Na-Cl gap at t1 (“1”), t1 corrected (“2”), t2 (“3”), t3 (“4”) and t4 (“5”)
